# American Journal of Ophthalmology

**Published:** 1863-04

**Authors:** 


### AMERICAN JOURNAL OF OPHTHALMOLOGY.

We have received the fourth number of this Journal, with the following Table of Contents:

*General Department.*—On Diphtheritic Conjunctivis, by De Graefe; The Anomalies of Mobility of the Human Eye; Art. III—Paralysis of the Abducens, by the editor.

*Special Department—Journalistic Reports.*—Contributions to the Knowledge of Glaucoma; Paper on Military Ophthalmia, read before the Academy of Medicine of Belgium; Analysis of One Hundred Ophthalmic Cases, showing the Comparative Frequency of the Various Diseases of the Eye in Hamilton, C. W.

*Bibliographical Reports.*—New Publications.

*Editorial Department.*—The Periodical International Congress of Ophthalmology; Notes, Queries and Replies.

